# Differential Metabolic Analysis of Rhizomes in Shancigu Based on Widely Targeted Metabolomics

**DOI:** 10.3390/metabo15100667

**Published:** 2025-10-13

**Authors:** Zhu-Yi Gao, Yi-Bo Yang, Li-Cheng Liu, Xue Li, Yan-Bo Huang, Qiang Zhou

**Affiliations:** 1 College of Biology and Environmental Sciences, Jishou University, Jishou 416000, China; 2 Shanghai Key Laboratory of Plant Functional Genomics and Resources, Shanghai Chenshan Botanical Garden, Shanghai 201602, China

**Keywords:** Shancigu, *Cremastra appendiculata*, *Pleione yunnanensis*, metabolomics

## Abstract

Background: Shancigu is a traditional Chinese medicine which is effective at clearing heat, detoxifying, dissipating masses, and resolving nodules. It consists of the dried pseudobulbs of orchids such as *Cremastra appendiculata*, or *Pleione yunnanensis*. To deeply understand the differences in the compositional and pharmacological active compounds in Shancigu, this study employed widely targeted metabolomics to analyze differential metabolites between two Shancigu species, *C. appendiculata* and *P. yunnanensis*. Methods: In this study, ultra-performance liquid chromatography–tandem mass spectrometry (UPLC-MS/MS) was used to qualitatively, quantitatively, and differentially analyze the metabolites of *C. appendiculata* and *P. yunnanensis*. Results: Metabolite profiling identified 2890 compounds across 13 classes. Within these, 687 metabolites showed significant differential abundance (23.76% total), including 331 upregulated and 356 downregulated compounds. Pathway enrichment analysis revealed these differential metabolites primarily concentrated in stilbenoid biosynthesis (types I and II) and flavonoid aglycone biosynthesis. The most highly expressed metabolites in the *Cremastra* group were L-tyrosine, dopamine and 3,4-dihydroxybenzaldehyde-xylose-glucoside, while in the *Pleione* group, the most abundant metabolites were 3,5-dihydroxy-2’-methoxy-4-methylbibenzyl, Shancigusin F and aloifol I. *C. appendiculata* preferentially accumulates flavonoids and phenolic acids whereas *P. yunnanensis* favors terpenoid and nucleotide derivative production. Conclusions: This study identifies key differential metabolites in *C. appendiculata* and *P. yunnanensis*, providing basic data for the overall evaluation and breeding of Shancigu, laying a foundation for further quality control and precise medication of Shancigu.

## 1. Introduction

Shancigu draws from five botanical families, including Orchidaceae, Amaryllidaceae, Menispermaceae, Liliaceae and Dioscoreaceae, with eight representative genera (*Cremastra*, *Pleione*, *Oreorchis*, *Tinospora*, *Iphigenia*, *Dioscorea*, *Lycoris*, *Tulipa*) yielding nine medicinally utilized species: *C. appendiculata*, *P. yunnanensis*, *P. bulbocodioides*, *O. patens*, *I. indica*, *D. persimilis*, *L. radiata*, *T. sinensis* and *D. bulbifera* [1]. The primary source of Shancigu is the dried pseudobulbs of *Cremastra appendiculata* (D.Don) Makino, commonly known as “Mao Ci Gu”. Two other Orchidaceae species, *Pleione bulbocodioides* (Franch.) Rolfe and *Pleione yunnanensis* Rolfe, also supply this material. These are collectively referred to as “Bing Qiu Zi”. Among these, *C. appendiculata* is the dominant species. The pseudobulbs from these plants are dried to produce the final medicinal product [2]. First documented in the Tang Dynasty Ben Cao Shi Yi (Compendium of Materia Medica), Shancigu exhibits sweet and slightly pungent flavors with a cool nature, targeting the liver and spleen meridians. It possesses functions for clearing heat, detoxifying, resolving phlegm, and dispersing nodules, and is clinically used to treat abscesses, scrofula, snakebites, and abdominal masses [3,4,5,6,7,8,9]. Shancigu contains over 190 bioactive compounds. Plants containing Shancigu synthesize phenanthrenes, dihydrophenanthrenes, bibenzyls, glycosides, terpenoids, anthraquinones, flavonoids and steroidal compounds, contrasting with substitute species producing colchicine, polysaccharides, flavonoids and starch [10,11]. These compounds demonstrate hypolipidemic and antigout effects, antioxidant and antimicrobial activities, neuroprotective properties, and antitumor actions mediated through inducing apoptosis, reducing inflammation, regulating oxidative stress [12]. Notably, phenanthrenes and bibenzyls exhibit cytotoxicity against diverse tumor cells (e.g., liver, gastric, colon, and breast cancers), establishing Shancigu as a key clinically used antitumor herb [13,14,15,16].

In recent years, with advancing research on natural medicinal plants, Shancigu has gained increasing scientific attention as a traditional herb with multifaceted pharmacological properties. Current studies primarily focus on nutritional profiling, structure–function characterization, mining of functional bioactive components, and extraction and identification of active compounds. These investigations have laid the groundwork for several important therapeutic applications, ranging from cancer treatment and infection control to anti-inflammatory interventions and neuroprotection. Despite this potential, metabolic differences among Shancigu species remain poorly understood. There is still limited research on systematic metabolomic characterization. Plant metabolomics can help resolve species-specific metabolite variation, thereby illuminating environmental adaptation mechanisms and medicinal compound biosynthesis pathways.

Widely targeted metabolomics provides a rapid and reliable method for detecting plant metabolites [17]. This approach integrates the advantages of both targeted and untargeted metabolomics, enabling rapid detection and quantification of diverse metabolites to assess metabolic differences underlying phenotypic variations. Currently, this metabolomic technique has been widely applied to study the diversity of and variation in secondary metabolites across different plant organs, cultivars, and species. Its accuracy has been demonstrated in analytical studies of various specialized secondary metabolites in tomatoes, tea plants, and other species [18,19]. Analysis focuses on characteristic metabolite signatures differentiating *C. appendiculata* and *P. yunnanensis* (Shancigu’s primary plant sources), using LC–MS as the core analytical platform augmented by statistical pattern recognition. Principal component analysis (PCA) coupled with partial least squares-discriminant analysis (PLS-DA) enabled chemical differentiation through integrated multivariate analysis. This methodology generates foundational evidence for bioactive nutrient research.

## 2. Materials and Methods

### 2.1. Plant Materials and Reagents

The experimental samples of Shancigu were collected from *C. appendiculata* and *P. yunnanensis* cultivated in the greenhouse of Shanghai Chenshan Botanical Garden (Shanghai, China). The plants were grown at 20–23 °C under a light intensity of 150 μmol of photons·m^−2^·s^−1^ with 14 h light/10 h dark cycle. Rhizome materials from 1-year-old plants were collected, immediately frozen in liquid nitrogen, and stored at −80 °C.

Methanol and acetonitrile (HPLC-grade) were supplied by Merck (Darmstadt, Germany), while acetic acid (analytical-grade) was procured from Aladdin (Shanghai, China).

### 2.2. Sample Preparation and Extraction

Samples were freeze-dried using a Scientz-100F lyophilization system. Lyophilized materials were homogenized with a cryogenic grinder (Retsch MM400, Haan, Germany). Prior to extraction, a precise amount (50.0 mg) of each lyophilized and homogenized plant powder was weighed to normalize for biological mass and ensure the starting material was consistent across all samples. Precisely 50 mg of powdered sample was mixed with 1200 μL of −20 °C pre-cooled 70% methanolic aqueous internal standard extract to correct for variations in extraction efficiency and instrument performance. The internal standard used was 2-chlorophenylalanine (purity: 98%; concentration: 1 ppm). The internal standard extraction solution was prepared by dissolving 1 mg of the standard compound in 1 mL of 70% methanol to obtain a 1000 μg/mL stock solution. It was then further diluted with 70% methanol to prepare a 250 μg/mL internal standard solution. The mixture underwent cyclic vortex mixing, followed by centrifugation. A 0.22 μm nylon filtration apparatus processed supernatants into HPLC-compatible vials to prepare them for UPLC-MS/MS characterization.

### 2.3. Metabolite Detection Parameters

The core analytical framework for experimental data capture integrates hyphenated UPLC-MS/MS apparatus as its principal detection modality. The chromatographic regime employed an Agilent SB-C18 analytical column (1.8 µm particle size, 100 mm × 2.1 mm dimensions). Chromatographic mobile phases were configured as follows: Eluent A, ultrapure water modified with 0.1% (*v*/*v*) formic acid; Eluent B, acetonitrile supplemented with 0.1% (*v*/*v*) formic acid. Elution gradient: The chromatographic gradient was programmed to initiate at 5.0% organic phase composition (t = 0.00 min), underwent a linear ramp reaching 95.0% (t = 9.00 min), and is held at 95% for 1 min. Commencing at 10.00 min, the organic phase proportion underwent a controlled descent to 5.0%, attaining the target concentration by 11.10 min. This baseline level persisted throughout the 2.90 min re-equilibration phase, concluding at 14.00 min. Chromatographic conditions is a 0.35 mL/min flow rate, 40 °C column temperature, and 2 μL injection volume.

Utilizing an electrospray ionization source thermally regulated at 500 °C, the mass spectrometric methodology applied distinct ion spray voltages of +5500 V operated in positive ion detection mode and −4500 V deployed under negative ion analysis conditions. Gas parameters were configured with Gas I at 50 psi, Gas II at 60 psi, and curtain gas at 25 psi, while collision-induced dissociation was operated in high-energy mode. Employing nitrogen as the collision gas maintained at medium pressure, multiple reaction monitoring (MRM) scanning was implemented throughout all analytical procedures. Rigorous optimization was performed on declustering potential and collision energy parameters to fine-tune each MRM transition, ensuring maximal detection sensitivity. Throughout each chromatographic segment, specific MRM ion pairs were dynamically monitored according to the elution profile of corresponding metabolites. Quality control (QC) samples, prepared by pooling aliquots from all experimental samples, were analyzed at regular intervals throughout the analytical sequence to monitor instrument stability and data quality.

### 2.4. Principal Component Analysis (PCA)

Unsupervised PCA was performed by statistics function prcomp within R package base package 3.5.1. The data was unit variance-scaled prior to unsupervised PCA.

### 2.5. Hierarchical Cluster Analysis (HCA)

The HCA results of samples and metabolites were presented as heatmaps with dendrograms. HCA was carried out by R package ComplexHeatmap 2.9.4. For HCA, normalized signal intensities of metabolites (unit variance scaling) are visualized as a color spectrum.

### 2.6. Differential Metabolites Selected

For two-group analysis, differential metabolites were determined by VIP (VIP >1) and absolute Log_2_FC (|Log_2_FC| ≥1.0). VIP values were extracted from the orthogonal partial least squares discriminant analysis (OPLS-DA) result, which also contained score plots and permutation plots; this was generated using R package MetaboAnalystR 1.0.1. The data was log-transformed (log_2_) and underwent mean centering prior to OPLS-DA. In order to avoid overfitting, a permutation test (involving 200 permutations) was performed.

### 2.7. KEGG Annotation and Enrichment Analysis

Identified metabolites were annotated using the KEGG Compound database (http://www.kegg.jp/kegg/compound/, accessed on 8 July 2024). Annotated metabolites were then mapped to KEGG Pathway database (http://www.kegg.jp/kegg/pathway.html, accessed on 8 July 2024).

## 3. Results

### 3.1. UPLC-MS/MS Metabolic Profile Analysis

**Figure 1:**
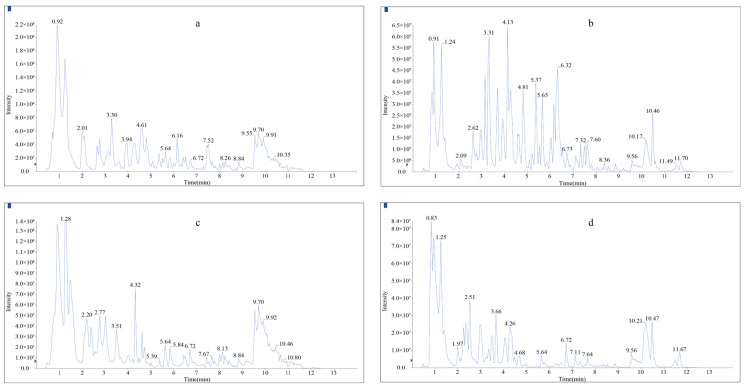
Total ion flow diagrams of *C. appendiculata* and *P. yunnanensis* Samples. (**a**) Positive ion mode of *C. appendiculata*. (**b**) Negative ion mode of *C. appendiculata*. (**c**) Positive ion mode of *P. yunnanensis*. (**d**) Negative ion mode of *P. yunnanensis*.

To investigate the metabolic diversity of Shancigu, a widely targeted UPLC-MS/MS analysis was performed on *C. appendiculata* and *P. yunnanensis* to elucidate differences in their secondary metabolites. The total ion current (TIC) chromatograms showed highly overlapping curves, indicating good signal stability during mass spectrometry detection (Figure 1). A comparative analysis revealed that the peak retention times of metabolites were highly consistent between the two Shancigu source species, suggesting no significant difference in the types of metabolites detected. In contrast, differences in peak intensity were observed between the two source species of Shancigu, indicating distinct variations in metabolite content between these different biosources.

### 3.2. Establishment of the Shancigu Metabolomics Database

**Figure 2:**
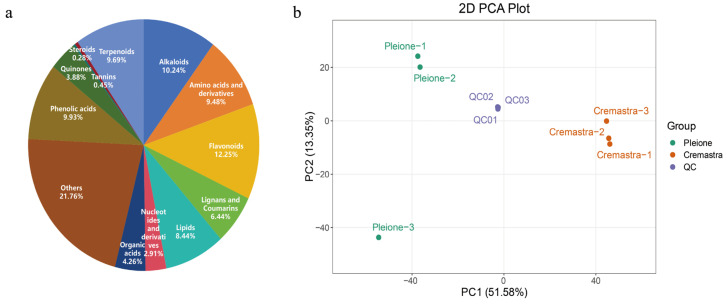
Detection and identification of metabolites. (**a**) Composition and proportion of metabolites across species. (**b**) Principal component analysis (PCA) of cross-species metabolic profiles.

As illustrated in Figure 2a, a total of 2890 metabolites were identified across *C. appendiculata* and *P. yunnanensis* samples (Supplementary Table S1). These metabolites were systematically classified into thirteen major categories, including alkaloids (10.24%), amino acids and derivatives (9.48%), flavonoids (12.25%), lignans and coumarins (6.44%), lipids (8.44%), nucleotides and derivatives (2.91%), organic acids (4.26%), phenolic acids (9.93%), quinones (3.88%), steroids (0.28%), tannins (0.45%), terpenoids (9.69%), and other compounds (21.76%). PCA analysis (Figure 2b) captured 64.93% total variance through its first two principal components (PC1: 51.58%, PC2: 13.35%). The PCA results revealed substantial metabolic divergence between *C. appendiculata* and *P. yunnanensis*, warranting further differential analysis. These findings underscore the rich diversity of secondary metabolites in Shancigu and highlight significant species-specific compositional variations.

### 3.3. Statistical Analysis of LC-MS/MS Analytical Results

**Figure 3:**
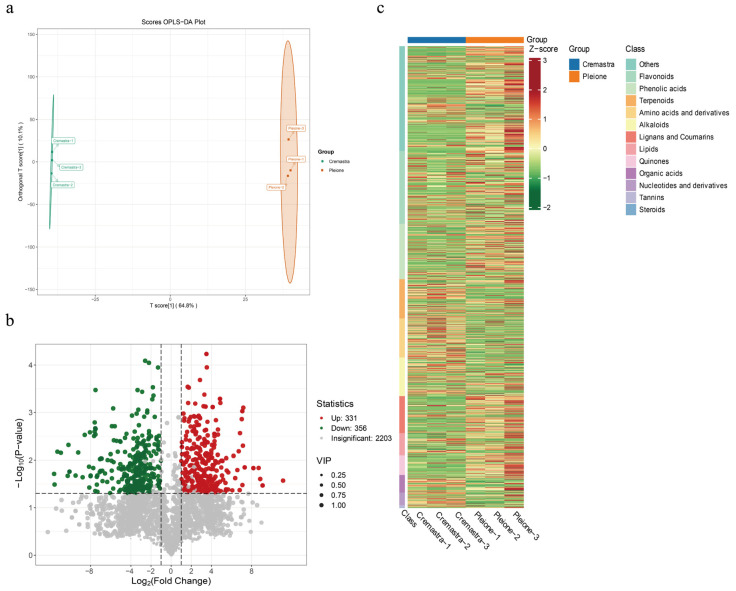
(**a**) OPLS-DA was used to analyze the differential metabolites. The model clearly segregates the samples from the two groups (Group *Cremastra* and Group *Pleione*). (**b**) Volcano plot of all detected metabolites. Red dots, green dots, and gray dots represent significantly upregulated, significantly downregulated, and insignificantly different metabolites, respectively. (**c**) Hierarchical clustering heatmap of the overall metabolite profiles. Rows represent metabolites and columns represent individual samples. The color scale indicates relative abundance levels, with red denoting high abundance and green denoting low abundance.

To eliminate the influence of other factors, supervised OPLS-DA was applied to the metabolite data of *C. appendiculata* (*Cremastra*) and *P. yunnanensis* (*Pleione*) (Figure 3a). The score plot showed that the T score and orthogonal T score components explained 64.8% and 10.1% of the total variance, respectively. The clustering of sample points within groups and clear separation between groups indicated good discrimination between the *Cremastra* and *Pleione* groups, demonstrating the model’s strong explanatory power and predictive capability. Based on the VIP, fold change (FC), and *p*-value screening criteria (Figure 3b), a total of 687 differential metabolites identified between *Cremastra* and *Pleione* 331 were significantly upregulated, while 356 were downregulated in *Cremastra* relative to *Pleione*. Additionally, 2203 metabolites showed no significant difference between the groups. Based on the comprehensive analysis, the final set of differential metabolites was further examined using a hierarchical clustering heatmap (Figure 3c), which clearly revealed distinct compound expression profiles between *Cremastra* and *Pleione*. Flavonoids and phenolic acids were predominantly expressed in *Cremastra*, whereas terpenoids, nucleotides and their derivatives were more highly expressed in *Pleione*, serving as characteristic high-abundance metabolite classes distinguishing the two groups.

### 3.4. Metabolic Pathway Enrichment Analysis

**Figure 4:**
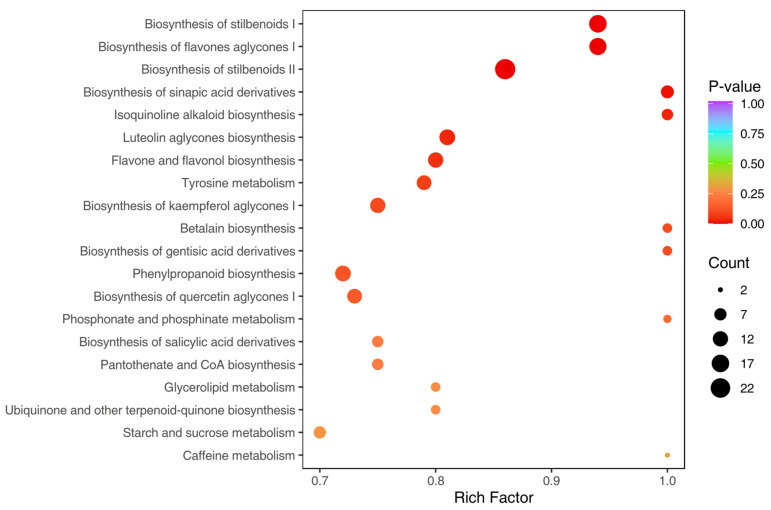
The top 20 enriched metabolic pathways in the Cremastra and Pleione samples. The size of each bubble reflects the number of metabolites associated with each pathway, with larger bubbles indicating a higher concentration of enriched metabolites.

Pathway enrichment analysis of the differential metabolites was performed using the KEGG database (Figure 4), revealing that *Cremastra* and *Pleione* shared enrichment in 20 key metabolic pathways. Most compounds were enriched in pathways such as biosynthesis of stilbenoid II, biosynthesis of stilbenoid I, and biosynthesis of flavone aglycone I. Biosynthesis of stilbenoid I and biosynthesis of flavone aglycone I exhibited the highest enrichment compounds and the lowest *p*-values, indicating that these pathways are highly active and statistically significant in the samples. The six significantly enriched pathways—biosynthesis of stilbenoid I, biosynthesis of flavone aglycone I, biosynthesis of stilbenoid II, biosynthesis of sinapic acid derivatives, isoquinoline alkaloid biosynthesis, and luteolin aglycone biosynthesis—cover a wide range of biological processes, including hormone regulation, nutrient transport, amino acid metabolism, and nucleic acid synthesis. Notably, the pathway related to stilbenoid biosynthesis suggests that stilbenoids play a central regulatory role in environmental adaptation in *C. appendiculata*, contributing to stress resistance, signal transduction, and potential health-promoting functions. The enrichment of the flavone aglycone biosynthesis pathway suggests that *C. appendiculata* might contribute significantly to antioxidant defense and stress resistance mechanisms. This finding raises the possibility that the species could play a key role in protecting cells from oxidative damage and enhancing resilience under environmental stress.

### 3.5. Identification and Screening of Differential Metabolites

**Figure 5:**
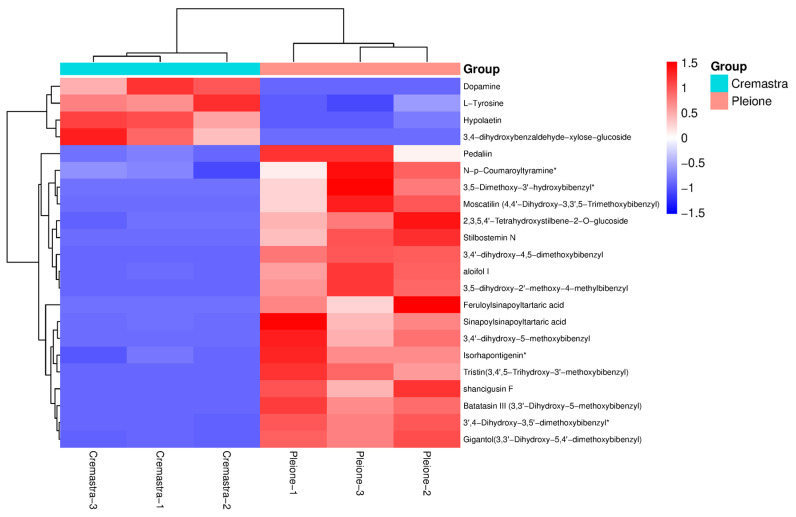
The hierarchical clustering heatmap of differential metabolites. The horizontal axis represents three biological replicates from the Cremastra group (Cremastra-1 to Cremastra-3) and three from the Pleione group (Pleione-1 to Pleione-3). Heatmap color coding corresponds to metabolite abundance levels (blue: low; red: high), where maximal red indicates peak expression in differential metabolites.

In this study, statistical screening defined key metabolites via VIP scores > 1 and adjusted *p*-values < 0.05. A total of 687 differential metabolites between the Cremastra and Pleione groups were selected for analysis. These metabolites included 83 phenolic acids, 63 terpenoids, 65 alkaloids, 63 amino acids and their derivatives, 69 flavonoids, 60 lignans and coumarins, 43 lipids, 16 quinones, 26 nucleotides and their derivatives, 28 organic acids, 1 steroid compound, 6 tannins, and 164 other types of compounds (see Supplementary Table S1). Based on the annotation information of the differential metabolites identified under these criteria, five significantly enriched metabolic pathways were selected for clustering analysis. The hierarchical clustering heatmap (Figure 5) revealed clear clustering and stratification effects between Cremastra and Pleione, with six metabolites showing the highest relative abundance: L-tyrosine, dopamine, 3,4-dihydroxybenzaldehyde-xylose-glucoside, 3,5-dihydroxy-2’-methoxy-4-methylbibenzyl, Shancigusin F and aloifol I. Among them, the most highly expressed metabolites in the Cremastra group were L-tyrosine, dopamine and 3,4-dihydroxybenzaldehyde-xylose-glucoside, while in the Pleione group, the most abundant metabolites were 3,5-dihydroxy-2’-methoxy-4-methylbibenzyl, Shancigusin F and aloifol I, as shown in Figure 4.

## 4. Discussion

This study employed a widely targeted metabolomics approach based on LC–MS/MS to analyze *C. appendiculata* and *P. yunnanensis*, revealing substantial differences in their metabolite compositions. PCA, OPLS-DA, and heatmaps were employed to compare *C. appendiculata* and *P. yunnanensis*, revealing significant differences in both the types and abundance of metabolites. Among the 2890 metabolites identified, a substantial portion comprised flavonoids, alkaloids, phenolic acids, and terpenoids, highlighting the remarkable metabolic diversity and complexity of *C. appendiculata*. Further analysis revealed 687 metabolites with significant differences in expression, including 331 that were upregulated and 356 that were downregulated.

KEGG pathway enrichment analysis revealed significant enrichment in several biosynthetic pathways, including biosynthesis of stilbenoid II, biosynthesis of stilbenoid I, biosynthesis of sinapic acid derivatives, isoquinoline alkaloid biosynthesis, luteolin aglycones biosynthesis, and biosynthesis of flavones aglycones I. These findings suggest that species-specific differences in secondary metabolism exert a notable influence, particularly in the biosynthesis of flavones aglycones I pathway, which is closely associated with medicinal properties and exhibited highly significant differences between species (*p* < 0.001), indicating potential substantial variations in pharmacological efficacy. Significant stilbenoid biosynthesis variations (*p* < 0.01) underlie species-specific resveratrol and piceid production differences. The distinct accumulation patterns of these specialized metabolites highlight the divergent chemical landscapes between the two species. Stilbenoid pathway variations generate radical-scavenging polyphenols, where STS catalysis yields resveratrol-type compounds. These compounds exhibit multifunctional bioactivities—including antioxidant and cardioprotective effects—establishing them as prime therapeutic candidates for natural product-derived therapies [20]. The differential regulation of these pathways provides a biochemical basis for the observed metabolic divergence and underscores the species-specific medicinal potential of each plant.

*C. appendiculata* featured abundant L-tyrosine, dopamine, and 3,4-dihydroxybenzaldehyde-xylose-glucoside metabolites reflecting its distinctive chemistry; *P. yunnanensis* expressed characteristic 3,5-dihydroxy-2’-methoxy-4-methylbibenz-yl, Shancigusin F, and aloifol I compounds.

L-Tyrosine serves as a crucial precursor for numerous pharmaceuticals, including levodopa and thyroxine. L-Tyrosine was identified as a key differential metabolite in our study, with L-tyrosine levels being significantly higher in *C. appendiculata*, consistent with its role as a central biosynthetic precursor. L-Tyrosine mediates neurological disorder treatment, mood regulation, and thyroid support, establishing its utility across therapeutic applications [21]. Additionally, L-tyrosine enhances flavor and color in food products, improving their sensory qualities [22].

The elevated dopamine content in *C. appendiculata* aligns with the active tyrosine decarboxylase (TYDC) and hydroxylation pathways observed in this species. The biosynthesis of dopamine in plants occurs through two primary pathways. One begins with the decarboxylation of tyrosine by TYDC to produce tyramine, which is then hydroxylated by monophenol hydroxylase (MH) into dopamine. The alternative pathway involves the hydroxylation of tyrosine by tyrosine hydroxylase (TH) to form L-dopa, which is subsequently decarboxylated by dopa decarboxylase (DD) to yield dopamine [23]. In plants, dopamine supports photosynthesis, improves CO_2_ utilization via stomatal regulation, and alleviates salt stress-induced inhibition of photosynthetic rates [24,25]. In medical contexts, dopamine enhances cardiac function and reduces post-asphyxia inflammatory myocardial injury [26].

3,4-Dihydroxybenzaldehyde-xylose-glucoside, a phenolic glycoside, may co-express with L-tyrosine and dopamine to balance plant growth and defense responses, while also detoxifying harmful aldehydes through glycosylation. On the other hand, 3,5-dihydroxy-2’-methoxy-4-methylbibenzyl—a structurally unique bibenzyl—displays a range of bioactivities such as antimicrobial, cytotoxic, and antiplatelet aggregation effects, indicating its potential for drug development [27].

Shancigusin F is a dihydrophenanthrene compound with a stilbenoid skeleton, showing antioxidant, anti-inflammatory, antitumor, antibacterial, antihypertensive, hepatoprotective, and lipid-lowering activities.

Aloifol I, a natural stilbene structural analog of resveratrol, exhibits antioxidant activity and NF-κB pathway inhibition. Its phenolic structure facilitates free radical scavenging and inflammation modulation while inhibiting tumor growth via PI3K/Akt and STAT3 signaling pathway regulation [28,29,30].

## 5. Conclusions

LC-MS/MS-based comprehensive metabolite profiling detected 2890 metabolites in *C. appendiculata* and *P. yunnanensis* pseudobulbs. Based on defined screening criteria, 687 significantly differential metabolites were identified, of which 331 were significantly upregulated in *C. appendiculata* compared to *P. yunnanensis*, while 356 were downregulated. Among these, the six most abundant metabolites across both species were L-tyrosine, dopamine, 3,4-dihydroxybenzaldehyde-xylose-glucoside, 3,5-dihydroxy-2’-methoxy-4-methylbibenzyl, Shancigusin F and aloifol I. KEGG enrichment analysis revealed six significantly enriched metabolic pathways: biosynthesis of stilbenoid I, biosynthesis of flavones aglycones I, biosynthesis of stilbenoid II, biosynthesis of sinapic acid derivatives, isoquinoline alkaloid biosynthesis, and luteolin aglycones biosynthesis. *C. appendiculata* preferentially accumulates flavonoids and phenolic acids, whereas *P. yunnanensis* favors terpenoid and nucleotide derivative production. Pharmacological objectives guide species selection, with *P. yunnanensis* advancing terpenoid/nucleotide-derived therapies and *C. appendiculata* serving flavonoid-based antioxidant/anti-inflammatory applications. This targeted strategy enhances Shancigu resource utilization precision and efficiency, boosting cost efficiency and key bioactive compound yields. These findings offer valuable insights for targeted breeding programs and informed species selection in the cultivation and utilization of Shancigu.

## Data Availability

The original contributions presented in this study are included in the article. Further inquiries can be directed to the corresponding author.

## Supplementary Materials

The following supporting information can be downloaded at: https://www.mdpi.com/article/10.3390/metabo15100667/s1. Annotated metabolite profiling data (Supplementary Table S1) provides a comprehensive list of identified metabolites, including their structural classifications, molecular formulas, quantitative levels across experimental replicates, and key statistical metrics. Additional identifiers and metabolic pathways are included where available. Compounds are categorized by chemical class and direction of change.
